# Bacteriological Profile and Antibiotic Susceptibility of Neonatal Sepsis Cases in the Neonatal Intensive Care Unit of a Tertiary Hospital in Türkiye

**DOI:** 10.3390/children11101208

**Published:** 2024-09-30

**Authors:** Bayram Ali Dorum, Şefika Elmas Bozdemir, Bensu Zadeoğlu Kral, Ayten Erdoğan, Salih Çağrı Çakır

**Affiliations:** 1 Division of Neonatology, Department of Pediatrics, Bursa City Hospital, Bursa 16110, Türkiye; 2 Division of Pediatric Infection, Department of Pediatrics, Bursa City Hospital, Bursa 16110, Türkiye; s.elmazbozdemir@saglik.gov.tr; 3 Department of Pediatrics, Bursa City Hospital, Bursa 16110, Türkiye; bensu.zadeoglu@sbu.edu.tr (B.Z.K.); ayten.erdoganordu@saglik.gov.tr (A.E.); 4 Division of Neonatology, Department of Pediatrics, Bursa Uludag University Faculty of Medicine, Bursa 16110, Türkiye; salihcagri@uludag.edu.tr

**Keywords:** coagulase-negative staphylococci, *Escherichia coli*, *Klebsiella* spp., neonatal sepsis

## Abstract

Objective: We aimed to determine the demographic data, mortality, and morbidity of early- and late-neonatal sepsis cases, the etiologic agents in these cases, and the antibiotic susceptibility of these agents. Methods: This study was conducted retrospectively in a tertiary neonatal intensive care unit (NICU). The demographic, clinical, and laboratory data of newborns diagnosed with culture-proven sepsis within 24 months were evaluated. Results: Two hundred and eleven culture data points belonging to 197 infants were evaluated. Forty percent of the infants had a history of premature birth. The most common clinical findings were respiratory distress and feeding intolerance. *Coagulase-negative staphylococci* (CoNS) were detected most frequently as early- and late-sepsis agents. The most common Gram-negative bacteria detected as late-sepsis agents were *Klebsiella* spp. and *Escherichia coli* (*E. coli*). The overall mortality rate was 10%. Conclusions: Neonatal sepsis continues to have high mortality rates in tertiary NICUs. CoNS was the most common agent, highlighting the importance of developing and maintaining personnel training and handwashing practices. It will be important to consider the resistance rates of *Klebsiella* spp., the most common Gram-negative agent in late-onset sepsis (LOS) cases, to commonly used antibiotics in empirical treatments.

## 1. Introduction

Despite advances in neonatal care, sepsis remains a major cause of mortality and morbidity in neonatal intensive care units (NICUs). Globally, infections are responsible for approximately 15% of neonatal deaths [1]. These rates are even higher in developing countries [2]. Cases in which the agent is detected in blood culture, along with clinical findings, are considered culture-proven sepsis cases. In early-onset sepsis (EOS), which occurs in the first three days of life, agents are microorganisms transmitted vertically or ascendingly from the mother or infected amniotic fluid. Maternal group B streptococcal (GBS) colonization or the presence of chorioamnionitis are important risk factors [3]. Intrapartum maternal fever and premature rupture of membranes are closely associated with early sepsis [4]. In late-onset sepsis (LOS), which occurs in later days, the agents cause infection through horizontal transmission from the environment or caregivers. Intensive care admission, interventions that disrupt skin integrity, catheter use, and invasive mechanical ventilation are the main risk factors [5]. The frequencies of both EOS and LOS increase inversely proportional to gestational age. Particularly in late hospital-acquired sepsis, the factors vary from unit to unit. Therefore, it is important to know the epidemiological data of the units and the antibiotic susceptibilities of the factors for the early and effective treatment of neonatal sepsis. Clinical symptoms in infected babies are very variable. Babies may present with different findings, including temperature instability, poor feeding, irritability, lethargy, tachypnea, tachycardia, hypotension, and shock [3]. Intrapartum fetal tachycardia, a low Apgar score, and meconium-stained amniotic fluid may be associated with early sepsis [4].

Due to its potentially devastating effects, physicians caring for newborns have a high suspicion and low diagnostic threshold for sepsis. This situation can sometimes lead to unnecessary and prolonged antibiotic use and increased antibiotic resistance [5]. This study aimed to evaluate the demographic, clinical, and laboratory data of infants diagnosed with culture-proven sepsis in a tertiary NICU. In addition, the antibiotic susceptibilities of microorganisms detected as EOS and LOS agents and the short-term outcomes of infants were shared.

## 2. Methods

This study was conducted retrospectively in the NICU of Bursa City Hospital. Our unit, which serves as an education and research hospital, is a 23-bed 3rd-level NICU. Approval was obtained from the hospital’s ethics committee for the study (No: 2022-12/8, Date: 5 October 2022).

Blood cultures were obtained from peripheral veins using a sterile technique. The skin was previously disinfected with 10% povidone-iodine and 0.5% chlorhexidine. Then, a 1 mL/kg blood sample was taken. The collected blood was injected into BACTEC Peds TM (Becton Dickinson, Ireland) culture bottles. Cultures were incubated and monitored in an automated system. Cultures with no growth signal during the seven-day follow-up were considered negative. Antibiotic susceptibility testing was performed according to the Clinical and Laboratory Standards Institute’s (CLSI) guideline (2020). The antibiotic resistance assay was performed using the disk diffusion method.

Culture-proven sepsis cases treated in the NICU over 24 months (January 2022–December 2023) were included in this study. The demographic data of the patients were obtained from the patient information registration system. Blood cultures were taken from all infants with clinical sepsis or sepsis risk factors before antibiotic treatment. The European Medicines Agency scoring is applied for sepsis assessment in our unit [6]. Ampicillin and gentamicin are applied as empirical treatments for EOS, and empirical cefotaxime and amikacin or vancomycin are applied for LOS. The clinical and laboratory data of the infants at the time of diagnosis were recorded. The clinical and laboratory results and antibiograms of patients with positive cultures included in the study were evaluated.

Statistical analysis of the data was performed using the IBM SPSS 28.0 (IBM Corp. Released 2021. IBM SPSS Statistics for Windows, Version 28.0. Armonk, NY, USA: IBM Corp.) statistical package program. The Shapiro–Wilk test was used to examine whether the data were normally distributed. According to the normality test result, if the continuous variables were normally distributed, the data were expressed as mean ± standard deviation, and if they were not normally distributed, they were expressed as median (minimum–maximum). In the comparisons of continuous variables between the two groups Student’s t-test was used if they were normally distributed and the Mann–Whitney U test was used if they were not normally distributed. Categorical variables were expressed as numbers and relevant percentage values. Pearson’s chi-square, continuity correction, and Fisher’s exact chi-square tests were used in the analysis of categorical data. The level of statistical significance was determined as *p* < 0.05.

## 3. Results

In a 24-month period, 1441 newborn babies were admitted to our clinic. Blood cultures were taken from 985 babies with suspicion of sepsis and antibiotic therapy was started. Treatment of 542 babies whose cultures did not show any signal of sepsis was ruled out during follow-up and terminated at the 48–72nd h. Neonatal sepsis was diagnosed in 443 babies. Of these, 211 culture data points belonging to 197 babies were evaluated. Of these, 43 babies were EOS (21.8%) cases and 154 babies were LOS (78.2%) cases.

Fifty-nine percent of our patients were male. Fifty-three percent were born prematurely. Premature birth, low birth weight, cesarean section, and low Apgar score were significantly more common in babies diagnosed with LOS. The most common clinical finding at the time of diagnosis was respiratory distress (37%). While mortality was 4.7% in EOS cases, this rate was 12.3% in LOS cases. The demographic and clinical data of the babies are given in Table 1.

C-reactive protein (CRP) values were significantly higher in late-sepsis cases (*p* = 0.002). The CRP value measured during culture-taking time was <10 mg/L in 44% of EOS cases at the time of initial diagnosis. This rate was 31% in the LOS cases (*p* = 0.131). Procalcitonin (PCT) was <2 ng/mL in 13% of LOS cases and was not evaluated as a diagnostic parameter in EOS cases. No significant difference was found between the groups in terms of white blood cell (WBC) and platelet (PLT) counts. Thrombocytopenia (<100,000) was detected in 14% of LOS cases and 1% of EOS cases (*p* = 0.457) (Table 2).

Fifty-seven percent (121/211) of the bacteria grown were Gram-positive. The most abundant bacteria were coagulase-negative *Staphylococcus* species (CoNS) (94, 44.5%). Eighty-eight (41.7%) of the isolated bacteria were Gram-negative. Fungi were isolated from two infants: *Candida tropicalis* in one patient and *Candida albicans* in the other. CoNS was again the most isolated causative agent in early-sepsis cases (24/43) and late-sepsis cases (70/154). The most isolated Gram-negative bacterial species in EOS cases was *Escherichia coli* (*E. coli*) (8/43), while it was *Klebsiella* spp. (32/154) in LOS cases (Table 3).

All CoNS bacteria were susceptible to linezolid and vancomycin, while 78% (74/94) were oxacillin-resistant. Gentamicin sensitivity was detected in 47.8% (45/94) of the CoNS bacteria. While 55% (5/9) of *Staphylococcus aureus* were oxacillin-resistant, all were vancomycin-sensitive. All streptococcal species were sensitive to oxacillin, vancomycin, and linezolid. Vancomycin resistance was detected in 20% (2/10) of enterococcal species and linezolid sensitivity was 100% for all Gram-positive bacteria.

All *E. coli* species were sensitive to meropenem and colistin. Of the Klebsiella spp., 73% (25/34) were resistant to cefotaxime, 32% (11/34) to meropenem, 59% (20/34) to piperacillin, 38% (13/34) to amikacin, 59% (20/34) to gentamicin, and 20% (7/34) to ciprofloxacin. *Stenotrophomonas maltophilia* isolated from three patients was sensitive to trimethoprim-sulfamethoxazole and levofloxacin. All were sensitive to colistin and tigecycline. No tigecycline resistance was observed for any of the pathogen agents. Other growth results and antibiotic resistance rates are given in Table 4.

Two fungal agents were detected in the blood cultures of two of our patients (1%). The *Candida* spp. grown did not have resistance to any known antifungals (fluconazole, amphotericin b, micafungin, voriconazole, and caspofungin).

## 4. Discussion

In this study, a significant number of culture-proven neonatal sepsis cases, the majority of which were LOS (78.2%), were evaluated. The demographic data, risk factors, agents, and antibiotic resistance of these agents were presented. This study aimed to increase the success of diagnosis and treatment in sepsis cases, which is a significant cause of mortality and morbidity. Because early diagnosis and the appropriate selection of antimicrobial agents are important to prevent sepsis-related neonatal deaths [7], it is critical to know the common agents and their antibiotic susceptibilities that form the basis of appropriate empirical antibiotic selection. It has been reported in the literature that appropriate empirical treatment selection shortens treatment durations and reduces vancomycin use [8,9].

In our study, the most important risk factors for neonatal sepsis were prematurity and low birth weight, which were present in more than half of the babies. It has been reported that premature birth increases the risk of sepsis by 3–10 times [4]. Schrag et al. [10] reported that there was 42.2% prematurity in their EOS cohort. Al-Matary et al. [11] found prematurity at 48.5% in EOS cases and 78.3% in LOS cases. Pan et al. reported prematurity as 76.2% in 202 LOS cases [12]. In a similar study, Pokhrel et al. [2] reported that 63.8% of culture-proven neonatal sepsis cases had low birth weight and 68.1% had a history of premature birth [2]. Similarly, in our study prematurity was found to be a statistically significant risk factor in LOS cases (*p* < 0.001).

In the study by Al-Matary et al. [11], 59.4% of sepsis cases were reported to be male. In a study by Pokhrel et al. [2], a 53.6% male predominance was reported. Similarly, 60% of our study cohort was male. However, this difference was not found to be statistically significant.

In the study by Al-Matary et al. [11], a low fifth-minute Apgar score did not show a statistically significant difference in LOS cases compared to EOS cases (*p* = 0.357). Similarly, in the study by Pokhrel et al. [2], a low fifth-minute Apgar score did not show a statistically significant difference between LOS and EOS cases. In our study, a low fifth-minute Apgar score and a history of need for resuscitation at birth were found to be significantly more common in LOS cases (*p* = 0.037, *p* = 0.006).

In the study of Al-Matary et al. [11], it was observed that there was no significant difference in premature rupture of membrane (PROM) history between EOS and LOS cases (*p* = 0.892), while in the study of Pokhrel et al. [2], PROM history was detected in 45% of cases and was found to be statistically significantly higher in EOS cases than in LOS cases (*p* < 0.0001). In our study, PROM history was detected in 6% of the cases and no statistically significant difference was found between the EOS and LOS cases.

In a study by Pokhrel et al. [2], the most common clinical finding was respiratory distress. Pan et al. [12] reported that the most common clinical findings in premature infants with sepsis were respiratory distress and feeding intolerance. When they evaluated all newborns, the most common findings were fever, respiratory distress, and jaundice. Similarly, in our study, the two most common findings were respiratory distress and feeding intolerance. Approximately 6% of our patients with late sepsis also had seizures. Pan et al. [12] reported this as 10.9%.

CRP is one of the most frequently used acute-phase reactants in the diagnosis and treatment response of neonatal sepsis. Al-Matary et al. [11] found CRP positivity in 33% of EOS and 84.1% of LOS cases. Pokhrel et al. [2] reported that CRP values were over 10 mg/L in 84% of patients in their study examining 69 neonatal sepsis cases. In our study, CRP values were found to be above 10 mg/L in more than half of the EOS cases and more than two-thirds of the LOS cases at the time of initial diagnosis.

Since PCT can physiologically increase in the first days after birth, it was not used as a diagnostic parameter in EOS cases in our unit. In Pan et al.’s [12] study, PCT values were found to be above 0.05 ng/mL in 98% of LOS cases. In our study, PCT values were found to be above 2 ng/mL at the time of diagnosis in 87% of our LOS cases.

The predictive value of WBC count in neonatal sepsis diagnosis is quite low. It is usually within normal values, even in definite sepsis cases. In Al Matary et al.’s [11] study, no significant statistical difference was observed between EOS and LOS cases in terms of WBC values. In our study, WBC values were found to be within normal limits in more than three-quarters of the patients, and no significant difference was found between the EOS and LOS groups.

Variations in PLT counts are important laboratory findings for neonatal sepsis. In Al Matary et al.’s [11] study, PLT values were found to be statistically significantly lower in LOS cases than in EOS cases (*p* = 0.003). In our study, although more PLT decreases were observed in LOS cases (16%) than in our EOS cases (1%), no statistically significant difference was detected.

Different studies have reported CoNS as the most common cause of neonatal sepsis in NICUs [13,14]. In developed countries, the most common EOS agent is reported as GBS [10]. Screening and prophylaxis programs against GBS, which is the most common EOS agent in developed countries, have caused a significant decrease in EOS cases due to GBS. However, Schrag et al. [10] showed in their approximately 10-year epidemiological study that GBS continues to be the leading EOS agent in the United States. Al-Matary et al.. [11] found GBS to be the most common EOS agent in a developing country, followed by *E. coli*. In our study, CoNS was the most common agent, while *E. coli* was the second most common. GBS was found to be the third most common EOS agent. GBS screening and prophylaxis are not routinely performed on pregnant women in Turkey. Pokhrel et al. [2] found *Klebsiella* spp. to be the most common agents in EOS cases, while CoNS was ranked second.

Regarding LOS agents, previous studies have shown that Gram-positive bacteria are the most common etiological agents and CoNS species are the most common organisms [15,16]. In developed countries, CoNS species continue to be the most common (53.2–77.9%) LOS agents [5]. In developing countries, the etiologic rate of CoNS in sepsis varies depending on region [17,18]. Guo et al. found that CoNS is the third most common organism responsible for LOS in southern China [19]. Al-Zahrani et al. found *Klebsiella* spp. to be the most common late-sepsis agents [20]. Al-Matary et al. [11] found *Staphylococcus* spp. to be the most common LOS agents, while *Klebsiella* spp. were the second most common agents. In our study, CoNS species were also the most common agents in late-sepsis cases. However, the most common Gram-negative agents in late-sepsis cases were *Klebsiella* spp. Al-Taiar et al. [21] found CoNS to be the most common agent and *Klebsiella* spp. to be the second most common agent of LOS in their study covering three different countries. Likewise, Gkentzi et al. [22] reported that CoNS and *Klebsiella* spp. were the most common LOS agents [22]. Pan et al.. [12] reported that Gram-negatives (*E. coli, Klebsiella pneumoniae*) were the most common agents in their LOS study, and CoNS was the third most common agent. These differences in the causative microorganisms isolated for both EOS and LOS may be due to differences in the design of the studies, the empirical antibiotic use policies of the units, and differences in compliance with hand hygiene practices, as well as differences in the social environment and country. Therefore, units need to evaluate their epidemiological data at certain intervals.

Fungi are important causes of late sepsis. However, their frequency and antifungal susceptibility vary among countries and units. Pan et al.. [12] reported that 5.9% of sepsis cases were due to *Candida* spp. In our study, only two patients had *Candida* spp. growth. No antifungal resistance was detected. In our unit, the administration of fluconazole at a dosage of 3 mg/kg two times a week is recommended in extremely low-birth-weight infants (<1000 g) by the Turkish Neonatal Society [4].

Pokhrel et al.’s [2] study showed that the majority of causative microorganisms develop resistance to cefotaxime and oxacillin, which are frequently used beta-lactam antibiotics. In the same study, it was determined that *Klebsiella* spp. showed resistance to colistin and tigecycline, albeit slightly. In our study, no resistance was detected to tigecycline, and tigecycline has not been used in our unit to date. While linezolid sensitivity was 100% among Gram-positive bacteria, vancomycin resistance was detected in *Enterococcus* growth in two of our patients.

Mortality was 10% when all cases were evaluated in our study. This rate, which was 4.7% in EOS cases, was 12.3% in LOS cases. This rate was 11% in Schrag et al.’s [10] EOS cohort and 16.8% in Pan et al.’s [12] LOS cohort. In Al-Matary et al.’s [11] study, these rates were 15.2% and 11.3% for EOS and LOS, respectively. We think that the low mortality rates in the EOS cases in our study are due to differences in etiologic agents. Schrag et al.. [10] reported that mortality was higher in *E. coli* sepsis cases and that *E. coli* was the cause of 51.5% of those who died from EOS. Al-Taiar et al. [21] found *Serratia* spp. to be the factor with the highest mortality rate (52%). In the same study, the factor causing the highest mortality in total was *Klebsiella* spp. In another study, mortality was reported as 16.8% in LOS cases [12]. The authors determined that *E. coli* was responsible for one-third of mortalities.

The retrospective and single-center natures of our study are the most important limitations. In addition, clinical and laboratory data could not be evaluated according to etiologic agents and gestational weeks. Neutrophil counts and total immature neutrophil rates were also not evaluated. In addition, maternal antibiotic use history could not be obtained. Along with these, the fact that the MIC values of resistant strains could not be presented was a possible limitation.

## 5. Conclusions

Neonatal sepsis continues to have high mortality rates in tertiary NICUs. Mortality is higher in LOS cases where prematurity and low birth weight are important risk factors. In this study, CoNS was found to be the most common causative agent, demonstrating the importance of developing and maintaining staff education and hand hygiene. It will be important to consider the resistance rates of *Klebsiella* spp., the most common Gram-negative agent in LOS cases, to commonly used antibiotics in empirical treatments.

## Figures and Tables

**Table 1:** Demographic and clinical data of the babies.

Variables, n (%)	EOS (N: 43)	LOS (N: 154)	Total (197)	*p*
Male gender, n (%)	26 (60)	92 (59)	118 (59)	0.999
Prematurity, n (%)	12 (27.9)	94 (61)	106 (53)	<0.001
Low birth weight, n (%)	12 (27.9)	89 (57.8)	101 (51)	<0.001
Cesarean section, n (%)	17 (39.5)	105 (68.2)	122 (61)	<0.001
Apgar score 5th min < 7, n (%)	5 (11.9)	42 (27.5)	47 (23)	0.037
Resuscitation at birth n (%)	5 (11.6)	49 (31.8)	54 (27)	0.006
Premature rupture of membranes, n (%)	3 (7)	9 (5.8)	12 (6)	0.787
Temperature instability, n (%)	11 (25.6)	23 (14.9)	34 (17)	0.115
Lethargy, n (%)	0	22 (14.3)	22 (11)	0.005
Respiratory distress, n (%)	13 (30.2)	60 (39)	73 (37)	0.385
Bleeding diathesis, n (%)	1 (2.3)	16 (10.4)	17 (8)	0.127
Irritability, n (%)	1 (2.3)	6 (3.9)	7 (3)	0.999
Cyanosis, n (%)	0	4 (2.6)	4 (2)	NA
Tachycardia, n (%)	4 (9.3)	16 (10.4)	20 (10)	0.999
Need for inotropes, n (%)	4 (9.3)	26 (16.9)	30 (15)	0.325
Feeding intolerance, n (%)	6 (14)	43 (27.9)	49 (24)	0.094
Hypotonia, n (%)	3 (7)	9 (5.8)	12 (6)	0.727
Convulsion, n (%)	0	9 (5.8)	9 (4)	0.210
Mortality, n (%)	2 (4.7)	19 (12.3)	21 (10)	0.261

**Table 2:** Laboratory data of the babies.

Variables, n (%)	EOS (N: 43)	LOS (N: 154)	*p*
CRP, mean (min–max)	3.49 (0.1–195)	17.15 (0.1–377)	0.002
PCT, mean (min–max)	5.7 (1–100)	2.44 (0.08–100)	0.429
WBC × 1000, mean (min–max)	12 (1.2–29.75)	12.55 (2–42.31)	0.273
PLT × 1000, mean (min–max)	246 (67–385)	295.5 (6–669)	0.117
CRP < 10 mg/L, n (%)	19 (44)	49 (31)	0.131
PCT < 2 ng/mL, n (%)	-	33 (13)	NA
Abnormal * WBC, n (%)	7 (16)	39 (25)	0.215
PLT < 100,000/mm^3^, n (%)	5 (1)	25 (16)	0.457

CRP: C-reactive protein, PCT: procalcitonin, WBC: white blood cell, PLT: platelet. * Normal white blood cell counts for EOS are between 6000 and 30,000/mm^3^, and are between 5000 and 20,000/mm^3^ for LOS.

**Table 3:** Distribution of microorganisms grown in blood cultures according to their species.

	EOS (43)	LOS (154)
Gram-positive bacteria		
*Coagulase-negative Staphylococci*	24	70
*Staphylococcus aureus*	1	8
*Streptococcus agalactiae*	6	-
*Streptococcus* spp.	-	2
*Enterococcus* spp.	2	8
Gram-negative bacteria		
*Klebsiella* spp.	2	32
*Escheria coli*	8	23
*Acinetobacter baumannii*	-	5
*Stenotrophomonas maltophilia*	-	3
*Enterobacter cloacae*	-	8
*Pseudomonas aeruginosa*	-	3
*Serratia marcescens*	-	4
Fungi		
*Candida* spp.	-	2

**Table 4:** Resistance rates of bacteria grown in blood cultures to commonly used antibiotics.

	Oxacillin	Vancomycin	Amikacin	Gentamicin	Ciprofloxacin	Cefotaxime	Meropenem	Colistin	Piperacillin	Levofloxacin
**Gram-positive bacteria**										
*Coagulase-negative Staphylococci*	74/94	0/94	28/94	43/94	26/94					
*Staphylococcus aureus*	5/9	0/9	ND	2/9	1/9					
*Streptococcus agalactiae*	0/6	0/6	ND	ND	ND					
*Enterococcus* spp.	ND	2/10	ND	8/10	ND					
**Gram-negative bacteria**										
*Klebsiella* spp.			13/34	23/34	7/34	25/34	11/34	0/34	20/34	4/34
*Escheria coli*			3/31	5/31	4/31	8/31	0/31	0/31	7/31	4/31
*Acinetobacter baumannii*			2/5	4/5	4/5	ND	3/5	3/5	5/5	5/5
*Enterobacter cloacae*			0/8	1/8	0/8	3/8	0/8	0/8	3/8	0/8
*Pseudomonas aeruginosa*			0/3	ND	1/3	ND	1/3	0/3	2/3	1/3
*Serratia marcescens*			1/4	1/4	0/4	1/4	0/4	4/4	1/4	ND

ND: No data.

## Data Availability

Necessary data are included within the manuscript. Explicit consent for the open sharing of the data was not obtained. The data are not publicly available due to privacy.
